# Caterpillars and Fungal Pathogens: Two Co-Occurring Parasites of an Ant-Plant Mutualism

**DOI:** 10.1371/journal.pone.0020538

**Published:** 2011-05-31

**Authors:** Olivier Roux, Régis Céréghino, Pascal J. Solano, Alain Dejean

**Affiliations:** 1 CNRS, Écologie des Forêts de Guyane (UMR-CNRS 8172), Campus Agronomique, Kourou, France; 2 CNRS, UMR 5245, EcoLab (Laboratoire d'Ecologie Fonctionnelle), Toulouse, France; 3 Université de Toulouse, UPS, Toulouse, France; 4 Aniane, France; Field Museum of Natural History, United States of America

## Abstract

In mutualisms, each interacting species obtains resources from its partner that it would obtain less efficiently if alone, and so derives a net fitness benefit. In exchange for shelter (domatia) and food, mutualistic plant-ants protect their host myrmecophytes from herbivores, encroaching vines and fungal pathogens. Although selective filters enable myrmecophytes to host those ant species most favorable to their fitness, some insects can by-pass these filters, exploiting the rewards supplied whilst providing nothing in return. This is the case in French Guiana for *Cecropia obtusa* (Cecropiaceae) as *Pseudocabima guianalis* caterpillars (Lepidoptera, Pyralidae) can colonize saplings before the installation of their mutualistic *Azteca* ants. The caterpillars shelter in the domatia and feed on food bodies (FBs) whose production increases as a result. They delay colonization by ants by weaving a silk shield above the youngest trichilium, where the FBs are produced, blocking access to them. This probable temporal priority effect also allows female moths to lay new eggs on trees that already shelter caterpillars, and so to occupy the niche longer and exploit *Cecropia* resources before colonization by ants. However, once incipient ant colonies are able to develop, they prevent further colonization by the caterpillars. Although no higher herbivory rates were noted, these caterpillars are ineffective in protecting their host trees from a pathogenic fungus, *Fusarium moniliforme* (Deuteromycetes), that develops on the trichilium in the absence of mutualistic ants. Therefore, the *Cecropia* treelets can be parasitized by two often overlooked species: the caterpillars that shelter in the domatia and feed on FBs, delaying colonization by mutualistic ants, and the fungal pathogen that develops on old trichilia. The cost of greater FB production plus the presence of the pathogenic fungus likely affect tree growth.

## Introduction

Mutualisms are interspecific interactions involving two or more species where each partner obtains resources that it would obtain less efficiently if alone, and so derives a net fitness benefit [1]–[3]. These mutualistic partnerships are transmitted from one generation to the next in one of two ways. In vertical transmission, hosts transmit symbiont offspring directly to their own offspring [4],[5], while in horizontal transmission the partners need to renew their association after each reproductive event [3],[6]. Biotic pollination, seed dispersal by animals, ant-plant associations and interactions between rhizobia or mycorrhiza and plant roots are transmitted horizontally and can be mutualistic [4],[6]–[9].

Myrmecophytes (or ant-plants) are involved in mutualisms with a limited number of so-called plant-ants that they shelter in domatia (i.e. hollow branches or thorns and leaf pouches) and usually provide with food through extra-floral nectaries (EFNs) and/or food bodies (FBs). In return, plant-ants protect their host plant from herbivores, competitors, encroaching vines and fungal pathogens [10]. Because the transmission of ant-plant mutualisms is horizontal, myrmecophytes have evolved several types of selective filters enabling them to host those ant species most favorable to their fitness [11],[12]. Host-plant selection by founding ant queens, for example, seems to be driven by chemical compounds [13]–[16]; however to enter into the domatia of certain myrmecophyte species, founding ant queens must be the right size or be able to recognize and to gnaw an entrance hole into the prostomata or thinner area, generally devoid of vessels [10],[17]–[19].

Nevertheless, these mechanisms do not keep the mutualism between myrmecophytes and plant-ants free from conflict, competition and/or exploitation by other ants or by non-ant species. In this context of competition for resources, the abilities of species are generally unequal, leading to hierarchically-organized systems with dominant and subordinate species. To survive, weaker competitors must develop colonization strategies, be resistant to perturbations, manage with fewer resources or have good longevity with the aim of conserving their access to the “niche” [20]–[22]. One alternative way to obtain an advantage over a better competitor is to be the first to obtain access to resources and to monopolize them. Often this advantage allows poor competitors to persist longer in habitats than they would otherwise [23],[24]. This phenomenon is known as “temporal priority” and has been documented in many taxa such as mycorrhizal fungi [25]–[27], plants [28],[29], amphibians [24],[30] and insects [23],[31].

It is well known that mutualistic plant-ant species compete for their host-plant [14],[32]–[34]. Moreover, mutualistic ants are not the only ones competing for this resource. Indeed, parasites of ant-myrmecophyte mutualisms-mostly ant species-are able to colonize the myrmecophytes, but do not provide them with protection [8],[9]. This was first shown for *Pseudomyrmex nigropilosus* that colonizes myrmecophytic *Acacia* and consumes their EFNs and FBs, but exhibits no defensive behavior [35]. Some non-ant insects are also able to colonize and parasitize myrmecophytes, benefiting from the shelter and food provided by the plant in different ways; for example, the larvae of the clerid beetle *Phyllobaenus* sp. parasitize myrmecophytic *Piper* trees, feeding on both the FBs and on mutualistic ants. The fitness of the host trees is reduced due to a greater investment in FB production and a decrease in biotic protection by the guest ants, with a subsequent increase in herbivory [36]. Also, females of the chrysomelid beetle *Coelomera* sp. open an entrance hole in the prostomata of *Cecropia* trees in the same manner as mutualistic *Azteca* ants. They then lay eggs in the domatia and the larvae feed on young leaves [37].

These insects, often less numerous and less aggressive than ants, must find weaknesses in the mutualism to be able to colonize their host tree and exploit its resources. Using chemical mimicry or camouflage to counter ant aggressiveness is one solution for getting past ant defenses [38],[39]; however, being the first to arrive to colonize new treelets might also represent a serious advantage because the biotic defense provided by mutualistic plant-ants is not yet in place and nearly leaves the plant without indirect defenses.

The myrmecophyte *Cecropia obtusa* (Cecropiacae), the focal species of this study, is mutualistically associated with several *Azteca* species (Dolichoderinae) whose founding queens and workers recognize the zone where the prostomata is situated and so establish colonies in the internodal domatia [13],[40]. In addition to shelter, the plant provides the *Azteca* colonies with food in the form of glycogen-rich Müllerian bodies produced by the trichilia, pads of dense trichomes situated at the base of the leaf petiole, and lipid-rich pearl bodies produced beneath young leaves. Mutualistic *Azteca* workers generally protect their host trees from defoliating insects, encroaching vines and fungal pathogens [17],[41]–[43], but this is not the case for saplings [44],[45].

Because the transmission of the *Azteca–Cecropia* mutualism is horizontal, the size of the internodes plus the production rate of the food bodies do not permit *Azteca* colonies to develop before the saplings reach ca. 1 m in height [44],[46]. Before *Cecropia* saplings reach this minimum size, herbivores and parasites may use this absence of mutualistic plant-ants to opportunistically take over the *Cecropia*'s resources; whereas, after this period of time, potential invaders must overcome the biotic defenses conferred by plant-ants [44].

We noted that recently-perturbed areas are rapidly occupied by thousands of *C. obtusa* saplings, permitting some caterpillars to live in the domatia and feed on the FBs, and that caterpillar presence was associated with the development of a fungus on the old trichilia. To broaden our understanding of the biological interactions and coexistence of these caterpillars within the *Azteca-Cecropia* mutualism, we conducted a correlation study where we posed the following questions. (1) Do caterpillars mainly or even exclusively feed on the FBs and, if so, does this activity increase FB production as is known for mutualistic plant-ants [36]? (2) Is caterpillar presence associated with the greater herbivory of *Cecropia* saplings? (3) Does this presence favor fungal development on the trichilia with deleterious consequences for the plant? (4) Can *Azteca* ants prevent colonization by caterpillars, or, inversely can caterpillars delay or even prevent colonization by ants thus allowing them to exploit *Cecropia* saplings longer?

## Materials and Methods

### Ethics Statement

This study was conducted according to relevant national and international guidelines.

### Study sites

**Figure 1:**
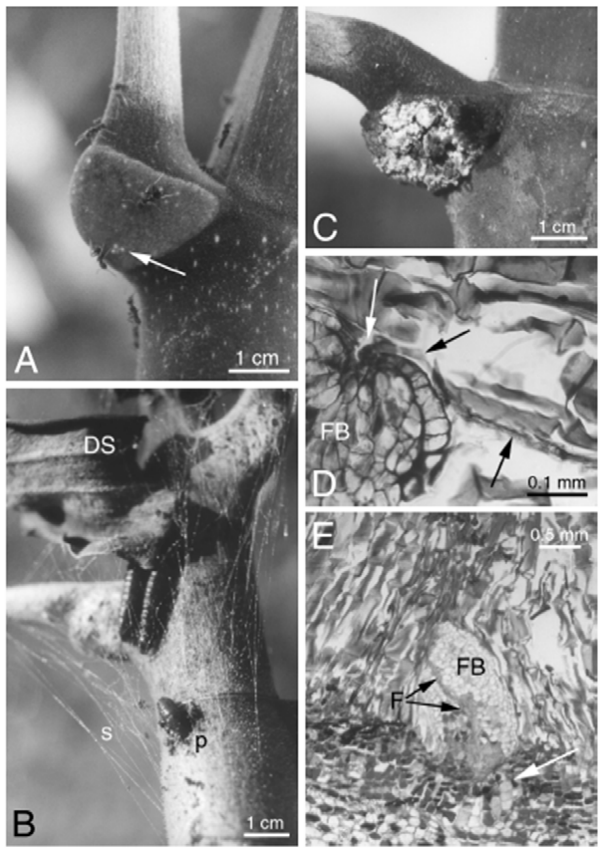
Trichilium of *Cecropia*. A- *Azteca alfari* workers removing food bodies (arrow) from a *Cecropia obtusa* trichilium. B- *Pseudocabima guianalis* caterpillars occupying the upper part of a *C. obtusa*. They gnawed an entrance hole into the prostomata (p) in order to enter into the hollow internodes. Secreted strands of silk cover the trichilium and keep the withered stipules (DS) from falling. C- Trichilium of *C. obtusa* infested by *Fusarium moniliforme*. D- Penetration by a filament of *F. moniliforme* (arrows) inside a food body (FB). E–*F. moniliforme* spreading throughout a food body (FB and black arrows). The cells of the trichilium at the base of the FB seem to react to the presence of fungus (white arrow).

We conducted this study between 2000 and 2009 in French Guiana near the *Petit Saut* dam (5°03′39″ N-53°02′36″W) and near the *Montagne des singes* (5°04′19.6″N-52°41′42.5″W). We selected and tagged *C. obtusa* that were ca. 1.15 m to 1.30 m tall (N = 610) between 2000 and 2006 in a cleared 1.5 ha zone situated near the dam, and on plants growing along a straight, recently-opened dirt road near the *Montagne des singes* (N = 64) between 2006 and 2009. In these areas, *C. obtusa* is mostly associated with *Azteca alfari* and *A. ovaticeps* whose colonies exploit Müllerian bodies (Fig. 1A), tend hemipterans in the host tree domatia and prey on insects landing on the leaves [42].

### The caterpillars' diet

To assess if the caterpillars feed exclusively on the FBs, in 2001 and 2002, we selected 83 saplings on which we had found at least one caterpillar. Preliminary studies conducted during a 24-hour period permitted us to learn that FBs are produced during the afternoon, between 15:00 and dusk (see also [47]–[50]), and that caterpillars are active during FB production. During a more comprehensive survey, we observed their behavior for 5 minutes per sapling in the morning between 8:00 and 11:00 as well as during FB production in the afternoon between 15:00 and 18:00. Observations were also made at night between 21:00 and 5:00 to ensure that the caterpillars were not active nocturnally.

### Food body production, herbivory, ant and caterpillar presence and tree growth

To quantify FB production, in 2003 we selected 30 saplings, each bearing at least five leaves: 10 sheltered *A. alfari*, 10 sheltered caterpillars and the remaining 10 were unoccupied. Because FB production increases when they are removed by ants or experimentally [17], we conducted experiments where both ants and caterpillars were prevented access to the upper zone of the trees where most of the FB production occurs. We did this by placing a ring of Tanglefoot® around the trunk under the lowermost leaf to isolate the top of each sapling, and thus prevent the ants and caterpillars from having access to the leaves and to the FBs. We then plugged the entrance holes situated in this upper, isolated section with a spot of Tanglefoot®. The ants and caterpillars could, nevertheless, move freely in and out of the domatia using the lower entrance holes giving them access to the lower part of the trunk and to older leaves with inactive trichilia. We placed aluminum foil shelters around the isolated trichilia to protect them from flying insects and to gather the FBs that dropped off [48],[51]. For each sapling and during 20 days, at ca. 19:00, we removed the FBs produced that day and that had fallen from the least mature trichilia onto the aluminum foil, and counted them. We compared the results using a repeated measures ANOVA followed by a Newman-Keuls' *post-hoc* test for multiple comparisons (GraphPad Prism 4.03 software).

We also tested, in 2003–2004, if the presence of ants and caterpillars affected the presence of defoliating insects by scoring the amount of herbivory on the oldest leaf on 90 saplings (30 sheltered *A. alfari*, 30 sheltered caterpillars, and the 30 others were unoccupied), each bearing at least five leaves. We chose the oldest leaves because they provide an idea of the history of the defoliation over the preceding ca. 18 months which corresponds to the lifespan of *C. obtusa* leaves [42]. We evaluated the percentage of foliar surface eaten by insects (FSE) using the following scale: (1) leaf intact; (2) slightly attacked: 0%<FSE≤25%; (3) somewhat attacked: 25%<FSE≤50%; (4) very attacked: 50%<FSE≤75%; and (5) extremely attacked: FSE>75%. We compared the results using the Kruskal-Wallis test.

To verify how caterpillars can delay or even prevent ant colonization and *vice versa*, every 8 months between June 2006 and June 2008, we noted which ant species or if caterpillars sheltered in the domatia and fed on the FBs on the 64 *C. obtusa* situated near the *Montagne des singes*. An additional survey was conducted in July 2009. In July 2008, we measured the height of the trees that had sheltered (1) *Azteca* colonies during the experimental period (*A. ovaticeps*: N = 10; *A. alfari*: N = 22), (2) neither ants nor caterpillars (N = 10), or (3) caterpillars during the entire experimental period or that had been replaced by an *Azteca* colony only during the last part of the experimental period (N = 14). All of these trees are the same age as they developed just after the dirt road was built near the *Montagne des singes* and have a similar exposure to the sun and to rain. We compared the results using an ANOVA and Newman-Keuls' *post-hoc* test.

### Fungal infestation of the trichilia

Between 2003 and 2005, we recorded the number of trees whose trichilia had a fungal infestation out of 610 *C. obtusa* saplings sheltering an *Azteca* colony (N = 349), caterpillars (N = 83), or not occupied (N = 178). We scored the presence *versus* absence of fungal infestation on the trees as “1” and “0”, respectively, and compared the results using the Kruskal-Wallis test and Dunn's *post-hoc* test.

To analyze how ant or caterpillar presence affected fungal development, we took samples of 20 trichilia with and 20 without developed mycelium from *C. obtusa* in all cases (i.e. trichilia taken from trees sheltering ants, caterpillars or unoccupied) and cultivated the mycelium in aseptic conditions in Sabouraud's nutritive substrate (N = 120 trichilia). We analyzed these samples under a microscope to verify how the mycelium develops on the trichilia. We first fixed the sampled trichilia with FAA (formalin, acetic acid, alcohol), and then embedded them in paraffin. We stained tissue sections with basic fuchsin light-green or toluidine blue contrasted with sodium molybdate.

Voucher specimens of the adult moths obtained after the metamorphosis of the caterpillars were identified as *Pseudocabima guianalis* (Lepidoptera, Pyralidae, Phyticinae) and were deposited at the Systematic Entomology Laboratory of the United States Department of Agriculture (USDA), Beltsville, Maryland. Fungal samples were identified as *Fusarium moniliforme* (Deuteromycetes) and were deposited at the *Laboratoire de biologie et taxonomie des microchampignons*, *Muséum National d'Histoire Naturelle*, Paris, France.

## Results

### The caterpillars

Observations made on the 83 saplings sheltering caterpillars permitted us to note that the first instar caterpillars lived under a silk shelter that they built between the stipules developing around the terminal bud, the trunk and the youngest leaf. They only left this shelter between 15:00 and 18:00 to feed on the FBs produced daily during that time period by the youngest trichilia, or sometimes by the other trichilia. As the shoot grows, the stipules, which normally drop off, are trapped by the silk (Fig. 1B; Fig. S1a). From their third instar, ca. 1.5-cm-long caterpillars, like ants, gnawed the prostomata in order to shelter in the last internode. They wove a silk shield above the upper part of the tree trunk and the youngest trichilium (Fig. 1B, Fig. S1b-d), and left the domatia only to feed on the FBs under the shelter of strands of silk. Pupation occurred inside the domatia. Larval and pupal development took about 30 days. Just-emerging moths leave the trunk by flying out through the stomata that are widened by the caterpillars when they are in their last larval stage.

### Fungal presence

**Figure 2:**
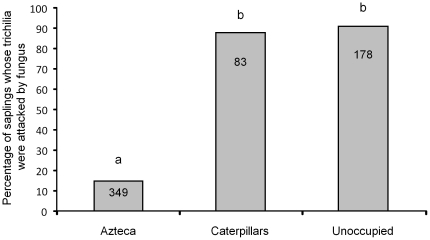
Trichilium infested by *Fusarium moniliforme*. Percentages of *Cecropia obtusa* saplings whose trichilia were attacked by *Fusarium moniliform*e in three situations: saplings sheltering an *Azteca* colony, saplings sheltering *Pseudocabima guianalis* caterpillars, and unoccupied saplings (N = number of saplings in each case). Statistical comparisons; Kruskal-Wallis test: H^3^_610_ = 338.1; P<0.001; Dunn's multiple comparison test, different letters indicate significant differences at P<0.001.

*Fusarium moniliforme* was present on 323 of the 610 *C. obtusa* saplings (53.0%), sometimes completely covering the trichilia (Fig. 1C). The percentage of infested individuals was significantly lower among saplings sheltering an *Azteca* colony than those sheltering *P. guianalis* caterpillars or not occupied, while the difference between the latter two cases was not significant (Fig. 2).

Normally, FBs are sub-spherical and homogeneous with reserve cells bordered by a cell wall and a thin cuticle. The penetration of the *F. moniliforme* mycelium into an FB occurs once it is already formed, but not necessarily completely developed. In Figure 1D, a filament of *F. moniliforme* can be seen entering into an FB. The bordering cells are in necrosis, as are the first reserve cells. The trichomes around the FB react strongly to the presence of the fungus, saturating their cell walls with lignin (Fig. 1E); whereas the FB cells show no reaction either in the cell wall or in the cytoplasm. Once inside the FB, the mycelium progressively invades all of the cells, down to the base of the FB (Fig. 1E). The first cells of the inner trichilium react to the presence of the mycelium when the FB is highly invaded. They seem to contain more tannin and are more elongated.

We noted a proliferation of the mycelium in *in vitro* cultures in Petri dishes with both healthy and infected trichilia, betraying the presence of spores in all cases (i.e. trichilia taken from trees sheltering ants, caterpillars or unoccupied).

### Ant and caterpillar occupancy of *Cecropia obtusa* trees

**Figure 3:**
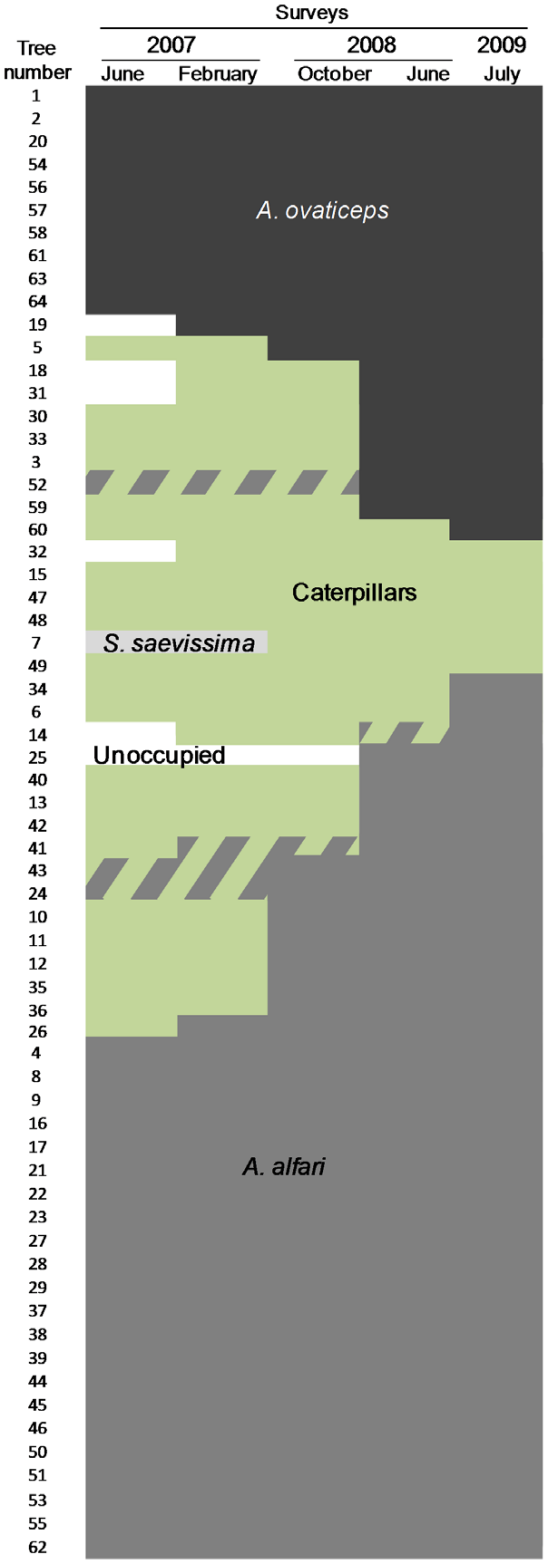
Host successional patterns for *Cecropia* treelets. Host successional patterns for each *Cecropia* sapling monitored during the 3-year survey on the dirt road near the *Montagne des singes*. Dashes correspond to trees sheltering both caterpillars and *A. alfari*. Trees were grouped to ensure the legibility of the figure, and so do not correspond to their geographic distribution.

Out of the 610 *C. obtusa* saplings studied near the *Petit Saut* dam, only 349 (57.2%) sheltered *A. alfari* or *A. ovaticeps* colonies. Among the others, 178 (29.2%) were totally unoccupied, while the remaining 83 (13.6%) sheltered three to six *P. guianalis* caterpillars at different larval stages (Fig. S1c). For the 64 *C. obtusa* surveyed during 3 years near the *Montagne des singes*, at the start of the survey the percentage of *C. obtusa* sheltering *P. guianalis* caterpillars was by far superior (39.1% or 25 trees out of 64; Fig. 3), illustrating that there are variations between areas. When present, caterpillars were also more numerous with some trees sheltering up to 12 caterpillars. Saplings were also associated with the two *Cecropia*-ant species typical of the area, *A. alfari* and *A. ovaticeps*, as well as, unexpectedly, the fire ant *Solenopsis saevissima* (tree N°7). Also, six trees were unoccupied at the start of the survey, and three of the 25 saplings bearing caterpillars were also occupied by *A. alfari* (trees N° 24, 43 and 52). This dual hosting was also observed later in the survey for two additional trees (trees N° 14 and 41), but after a few months, all five trees were occupied only by *Azteca* colonies. Note that, in the end, tree N° 52 was colonized by *A. ovaticeps.* Over the course of the different surveys, unoccupied trees were colonized by caterpillars (four cases) or directly by *Azteca* ants (trees N° 19 and 25). Although *S. saevissima* workers exploited the FBs and were aggressive towards flying insects landing on their host tree foliage, tree N°7 was colonized in the end by caterpillars (Fig. 3). While the two *Azteca* species occupied more and more trees over time, the number of trees sheltering *P. guianalis* caterpillars first increased and then decreased. They were replaced by *A. alfari* or *A. ovaticeps* colonies on 15 and 10 trees, respectively. At the end of the experiment-so 3 years after the beginning of the survey-six trees still sheltered caterpillars. During this entire lapse of time, the trees occupied by *A. alfari* or *A. ovaticeps* were never colonized by caterpillars. In July 2009, four *Cecropia* trees still sheltered caterpillars.

### Food body production, herbivory and tree growth

**Figure 4:**
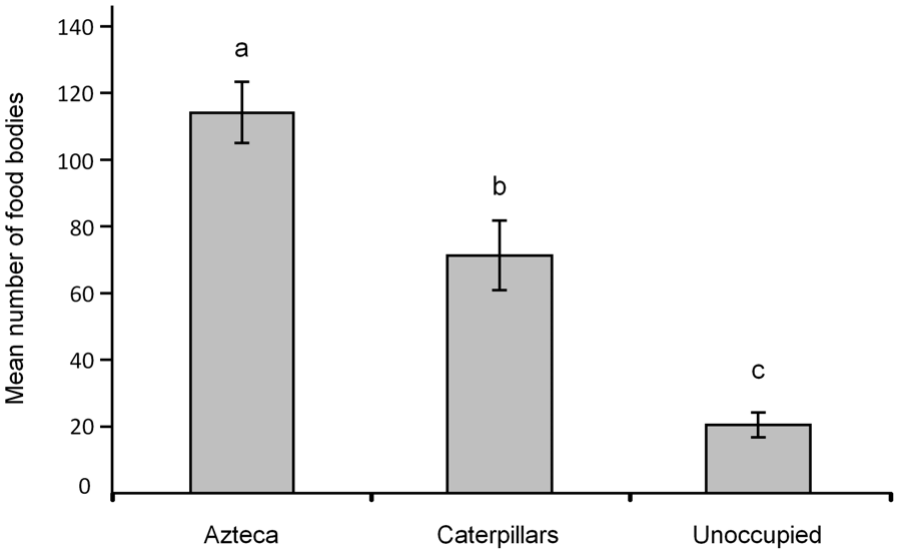
Food body production. Comparison of the mean food body production per leaf and per day (±SE) by the youngest trichilia on *Cecropia obtusa* saplings during 20 successive days in three situations: saplings sheltering an *Azteca alfari* colony, saplings sheltering *Pseudocabima guianalis* caterpillars, and unoccupied saplings (10 individuals in each case). Statistical comparisons; repeated measures ANOVA: F^2^_30_ = 64.81; P<0.001; Newman-Keuls' *post-hoc* test: different letters indicate significant differences at P<0.001.

FB production was significantly higher for saplings sheltering *Azteca* ants than for those sheltering caterpillars and for the latter compared to unoccupied saplings (Fig. 4).

**Figure 5:**
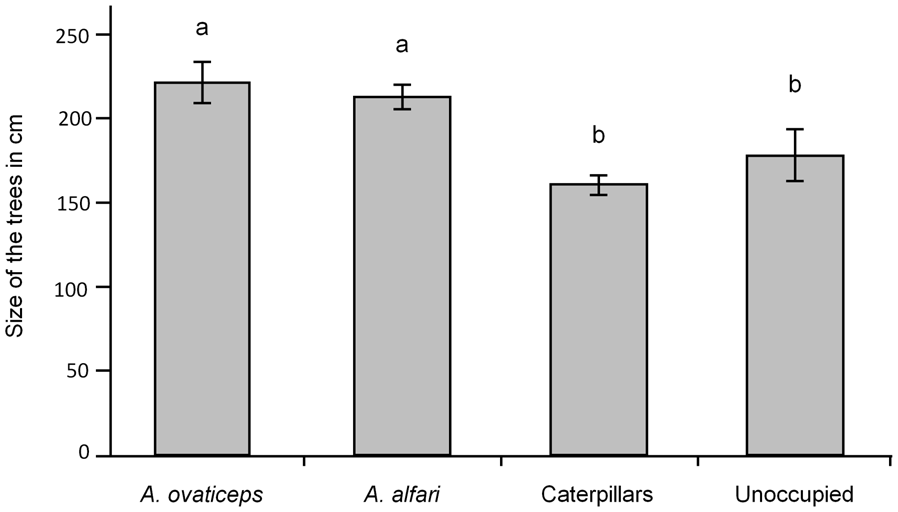
Height of *Cecropia* treelets. Mean height of trees (±SE) that sheltered colonies of one of the two *Azteca* species, *Pseudocabima guianalis* caterpillars, or that sheltered neither *Azteca* nor caterpillars during the experimental period. Statistical comparisons (normality and equal variance tests passed); ANOVA: F^2^_56_ = 8.56; P<0.0001. Newman-Keuls' *post-hoc* test: different letters indicate significant differences at P<0.05.

We did not note significant differences in the percentage of foliar surface eaten by defoliating insects between the *C. obtusa* sheltering an *Azteca* colony, caterpillars, or not occupied by either ants or caterpillars (Kruskal-Wallis test, H^2^_90_ = 1.813; P>0.05). Nevertheless, caterpillar presence affected tree growth as those sheltering *Azteca* colonies during this experimental period were significantly taller at the end of the survey than those sheltering caterpillars or those that were unoccupied (Fig. 5). The differences were not significant between trees sheltering colonies of the two *Azteca* species, or between trees sheltering caterpillars or that were unoccupied.

## Discussion

All in all, these results constitute a new step in understanding the nature of the parasitism of the Azteca-*Cecropia* mutualism by non-ant insects. Like *Coelomera* chrysomelid beetles [37] and mutualistic ants, *P. guianalis* caterpillars at their third larval stage recognize the prostomata and gnaw an entrance hole to shelter in the host tree domatia. The difference with the damage caused by *Coelomera* is that it is direct as they feed on young leaves [37], whereas *P. guianalis* caterpillars, like mutualistic ants, feed on the FBs produced by the plant. They are indirectly detrimental to their host tree because they allow *Fusarium* to develop on the trichilium. Moreover, although it is possible that female moths select the most productive trees for their offspring [52], the caterpillars seems to induce an increase in FB production, as do *Azteca* workers and clerid beetle (*Phyllobaenus* sp.) larvae on *Piper* [36]. However, unlike some mutualistic ants [53], *P. guianalis* caterpillars do not provide any services in return for this increase in FB production.

This type of increase in FB production is usually stimulated when mutualistic ants remove the FBs because the space thus made available reduces the pressure on the trichilia and favors the production of the next group of FBs [17],[54]. In the absence of mutualistic ants, FB production remains low, but is high enough to be attractive to founding ant queens [17],[54]. Because our experimental design did not allow *Azteca* ants, caterpillars or other insects access to the trichilia, FB production should have been reduced to the same level as on unoccupied trees. Yet, this was not the case, suggesting that a factor other than FB removal plays a role, such as the plant obtaining nutrients from its ant or caterpillar inhabitants [55].

When parasitic ants are present, the establishment of mutualistic species is durably prevented, and plant fitness is lessened due to increased herbivory [8]. The presence of non-ant parasites does not imply the exclusion of mutualistic ants, but host tree leaves can suffer herbivory if these insects feed on the plant [37] or on plant-ants that are therefore unable to protect their host trees [36]. However, here, the presence of *P. guianalis* caterpillars was not associated with greater herbivory compared to trees sheltering *Azteca* colonies or unoccupied trees because saplings rely on secondary anti-defoliator compounds and structures for their protection [37],[44],[45].

Even though the saplings did not suffer greater herbivory, the protective mutualism is very disrupted as, when present, *Azteca* colonies significantly limit the development of the *Fusarium* mycelium. Indeed, we show that in the absence of mutualistic ants, *Fusarium* developed on the trichilia of both unoccupied trees and trees sheltering caterpillars. Moreover, this fungus is known to produce growth-inhibiting mycotoxines that are also responsible for necrosis in plants [56],[57] and insects [58],[59]. Consequently, likely due to the presence of this pathogen plus the cost of producing FBs, the growth rate of the trees that sheltered caterpillars during the survey conducted at the *Montagne des singes* was affected if compared to those that sheltered *Azteca* colonies during the same period (Fig. 5).

Therefore, mutualistic *Azteca* likely control the extent of the fungal infection in the same way that, by defending myrmecophytic *Piper* from stem-boring insects, *Pheidole* ants reduce fungal infections [60]. On the other hand, when deprived of their mutualistic *Crematogaster* ants, myrmecophytic *Macaranga* suffer from both shoot borers and pathogenic fungi [61]. Indeed, ants' antifungal activity is well known [37],[60],[62] and can be due to chemicals produced by the venom, the metapleural or the mandibular glands [63]–[66] or results from the activity of symbiotic bacteria [67]. On the plant side, it has been noted that some myrmecophyte species have lost their intrinsic physiological defenses against fungal infection [60],[68]. The spores of *Fusarium* can be disseminated by both wind and insects, particularly Lepidoptera larvae that are resistant [58], explaining why the *P. guianalis* caterpillars were not infected by *Fusarium*, while the host plant trichilia were. Because we did not note a difference in the amount of herbivory between ant-inhabited and ant-free *Cecropia*, one can hypothesize that *Fusarium* might be the main selective driving force in the present situation. In that case, the earlier the *C. obtusa* treelets shelter mutualistic *Azteca* colonies, the more they will grow due to the antifugal activity of the ants (particularly by suppressing spore germination [69]). Later, as the trees grow and their ability to synthesize secondary antiherbivore compounds lessens, *Azteca* workers, that belong to larger and larger colonies, will take over and provide their host trees with biotic protection from herbivorous insects [11],[17],[42].

We cannot exclude that the occupancy by caterpillars could be favored by the plant's characteristics or micro-environmental conditions rather than by the competitive and colonizing abilities of the insects. Nevertheless, because *C. obtusa* is a pioneer species that develops in large numbers in recently cleared areas, some insect species with a high rate of dispersal can be the first to reach the resources provided by the trees (see [51] for Reduvidae feeding on *C. obtusa* FBs before the installation of *Azteca* colonies). It is thus likely that a temporal priority enabled *P. guianalis* caterpillars to install themselves on certain trees prior to the arrival of the plant's mutualistic *Azteca* ants with which they are involved in competitive exclusion (see also [23],[24] for a temporal priority concerning ant-myrmecophyte mutualisms).

When caterpillars do successfully colonize a tree, several overlapping generations can be observed-the youngest sheltering under the stipules developing around the terminal bud, the trunk and the youngest leaf, and the oldest in the host tree domatia. During the hours of FB production, both young and old caterpillars share the FBs on the trichilia. Smaller caterpillars likely benefit from the silk woven above the trichilia by larger mates as protection from competing ants, predators and/or parasitoids which seem repelled, and so do not walk on it (pers. obs.). The overlap between different generations of caterpillars plus the fact that certain trees can be occupied during several years imply that female *P. guianalis* moths lay eggs on trees already sheltering caterpillars.

When an incipient *Azteca* colony successfully colonizes their host tree, large caterpillars seem to deny the first workers access to the most productive trichilia by weaving silk above them. They, thus, indirectly slow down colony growth as these foraging workers only have access to the lower, less-productive trichilia often already covered by *F. moniliforme*. Consequently, the development of the colonies depends mostly on the ants attending hemipterans in the internodes of the host trees (if any). So, although caterpillars can delay *Azteca* colonization, the *Cecropia* trees are finally exclusively occupied by *Azteca* ants. Furthermore, once *Azteca* colonizes a *Cecropia* tree, the workers exploit the FBs on the upper, most-productive trichilia and patrol the foliage, rendering the situation irreversible by preventing colonization by caterpillars. Indeed, the *Azteca* workers, that are able to capture insects the size of a female *P. guianalis* moth [42], probably destroy any insect eggs that have been successfully laid on their host plant's foliage (see [70] and references therein).

In conclusion, *P. guianalis* caterpillars are able to “break the code” [36] of the *Azteca-Cecropia* mutualism by recognizing the prostomata and exploiting the resources *Cecropia* normally supplies to mutualistic *Azteca*; they even induce greater FB production. Although no higher herbivory rates were noted, these caterpillars are ineffective in keeping a fungus from developing on the trichilia of their host trees, something that mutualistic *Azteca* ants can do. By denying mutualistic ants access to FBs and young leaves, *P. guianalis* caterpillars become a more formidable competitor of mutualistic ants and so are parasites of both *Cecropia* saplings and the *Azteca-Cecropia* mutualism.

## Supporting Information

Figure S1 **Caterpillars on** ***Cecropia*** **treelets.** **a** Upper part of a young *Cecropia obtusa* sheltering *Pseudocabina guianalis* caterpillars. Strands of silk produced by the caterpillars keep the stipules of two leaves against the trunk (yellow arrow). An entrance hole gnawed by a caterpillar is visible (white arrow). Note that the leaves were not attacked by defoliating insects. **b** A forth instar caterpillar eating food bodies on the youngest *trichilia* on a tree, some strands of silk are visible. **c** Three larval stages eating food bodies on the same *trichilia*. **d** A forth instar caterpillar eating food bodies on a *trichilia* that began to be infected by *Fusarium moniliforme*. **e** Several caterpillars at different stages on a *trichilia*, some strands of silk are visible.
(TIF)
