# Supplementary figures and images for: Caterpillars and Fungal Pathogens: Two Co-Occurring Parasites of an Ant-Plant Mutualism
Source: PLoS One. 2011 May 31;6(5):e20538. doi: 10.1371/journal.pone.0020538 (PMC3105098; doi:10.1371/journal.pone.0020538)

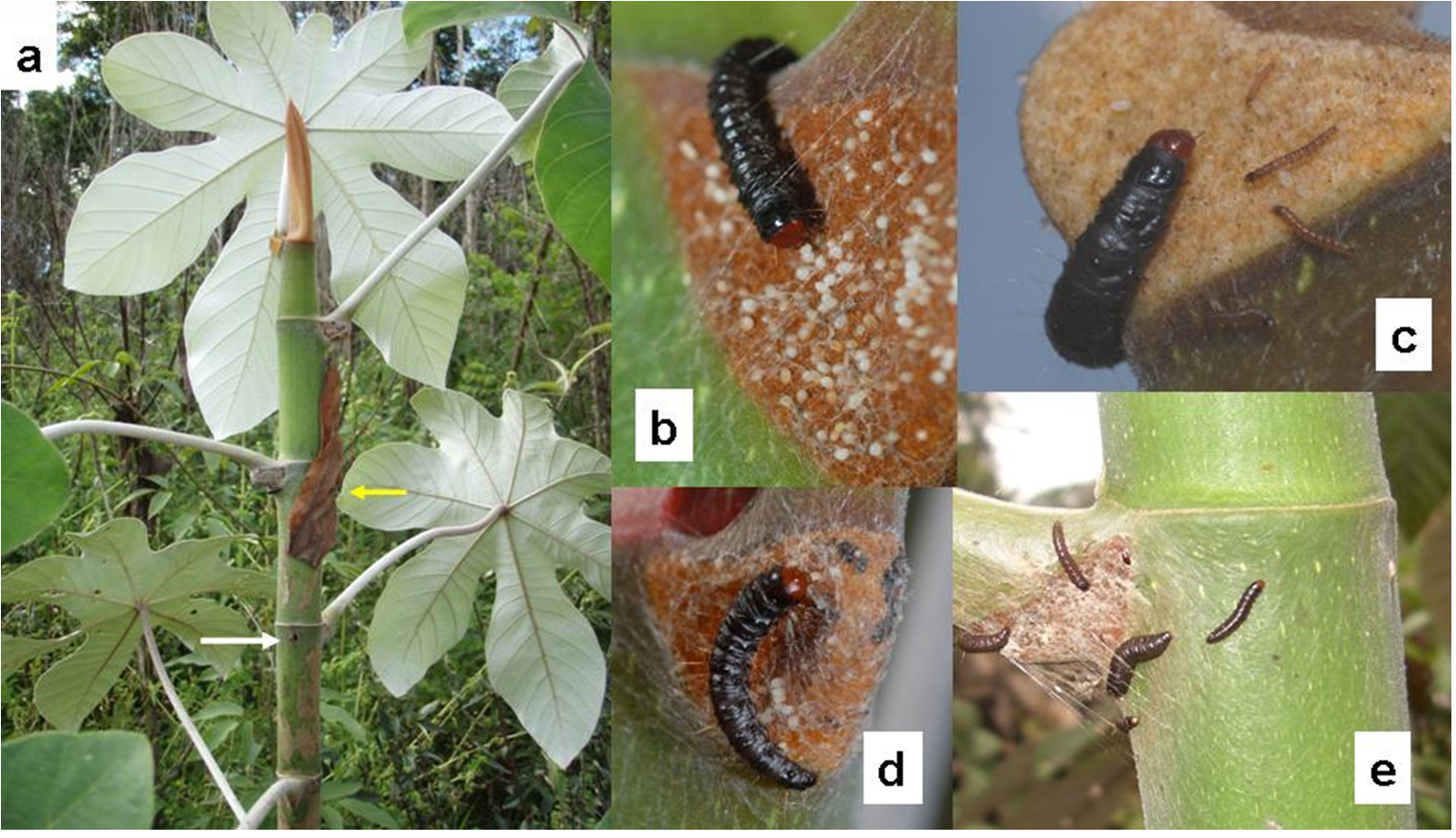

Supplement: Figure S1 — Caterpillars on Cecropia treelets. a Upper part of a young Cecropia obtusa sheltering Pseudocabina guianalis caterpillars. Strands of silk produced by the caterpillars keep the stipules of two leaves against the trunk (yellow arrow). An entrance hole gnawed by a caterpillar is visible (white arrow). Note that the leaves were not attacked by defoliating insects. b A forth instar caterpillar eating food bodies on the youngest trichilia on a tree, some strands of silk are visible. c Three larval stages eating food bodies on the same trichilia. d A forth instar caterpillar eating food bodies on a trichilia that began to be infected by Fusarium moniliforme. e Several caterpillars at different stages on a trichilia, some strands of silk are visible. (TIF) [file pone.0020538.s001.tif]
